# Psychometric properties of the Iranian version of Diabetes Empowerment Scale (IR-DES-28)

**DOI:** 10.1186/2251-6581-11-4

**Published:** 2012-08-02

**Authors:** Mohammad Yoosef Mahjouri, Seyed Masoud Arzaghi, Ramin Heshmat, Patricia Khashayar, Ensieh Nasli Esfahani, Bagher Larijani

**Affiliations:** 1 Endocrinology and Metabolism Research Center (EMRC), Tehran University of Medical Sciences, Tehran, Iran; 2 Endocrinology and Metabolism Research Center, Shariati Hospital, Kargar St, Tehran, 14114, Iran

**Keywords:** Diabetes, Diabetes empowerment, Diabetes empowerment scale, Psychometric properties

## Abstract

**Background:**

This study was designed to determine the primary psychometric properties of the Iranian version of the Diabetes Empowerment Scale (IR-DES-28).

**Methods:**

After translating the questionnaire into Persian, re-translating it into English and obtaining the confirmation of the diabetes specialists regarding the accuracy of the translated questionnaire, 100 patients with type 2 diabetes selected using a systematic random sampling method completed the Iranian-DES-28. The validity of IR-DES-28 was evaluated by construct, concurrent and criterion validity, whereas its reliability was assessed by test re-test, internal consistency and splitting method.

**Results:**

Psychometric analysis confirmed the reliability and validity of the IR-DES-28 in three subscales: Self-awareness and managing psychological aspects of diabetes (α=0.80), goal achievement ability (α=0.50), the ability of setting goals (α=0.79). Test-retest reliability, evaluated through Pearson coefficient correlation, was equal to 0.74 (P <0.01). The total score of IR-DES-28 and that of the extracted subscales were correlated with HbA_1c_ levels (metabolic control) (P <0.01). The findings also supported the concurrent validity of the questionnaire.

**Conclusion:**

The validity and reliability of the Iranian-DES scale is acceptable. Thus, the scale could be an appropriate instrument in evaluating the empowerment-based education programs.

## Introduction

Diabetes mellitus is one of the most serious illnesses, nowadays afflicting many people in Iran and other countries. Diabetic patients must take a series of daily decisions regarding nutrition, physical activity, medication, blood glucose monitoring, and stress management. They must also interact effectively with the health care system, their family members, and friends to obtain the support needed to manage and control their disease [1]. The education programs in diabetic patients aim to improve the patients’ ability to obtain the self efficacy required to gain control over daily self-care [2,3]. Anderson et al. described that self efficacy can be gained through the adaptation of an empowerment approach, adding that such a technique has a significant and positive effect on the patient’s metabolic control and quality of life. They explained that empowerment philosophy is based on the assumption that to be healthy, people need to have the psychosocial skills required to change their personal behavior, social situations, and the institutions that influence their lives. These skills can play an important role in the development and fulfillment of effective and successful diabetes self care program [4]. Therefore, empowerment is a strategy and process through which people obtain greater control over their decisions and actions related to their health and diabetes management [5]. In other words, empowerment in this area is defined as helping diabetic patients discover and develop a natural capacity and ability to become responsible for one’s own life [6]. Previous researches have indicated that empowering the patients through providing them with appropriate and sufficient education programs can increase their knowledge about diabetes and promote their collaboration with physicians [6-9].

An appropriate tool is needed to evaluate these empowerment education programs. "Diabetes Empowerment Scale (DES)" is one of the main instruments used in this regard. The first version of the US-DES was a 37-item questionnaire with eight subscales, including satisfaction and dissatisfaction related to living with diabetes; identification and achievement of personally meaningful goals; application of systematic problem-solving process; coping with emotional aspects of living with diabetes; stress management; appropriate social support; self-motivating; and making cost/benefit decisions regarding behavior changes [4,10]. The assessment of this first version indicated that only three of these eight subscales has an internal consistency of greater than 0.80, thus the authors reduced the questionnaire from 37 to 28 items with three subscales, including managing the psychosocial aspects of diabetes (α=0.93), assessing dissatisfaction and readiness to change (α=0.81), and setting and achieving diabetes goals (α=0.91). The reliability and validity of the recent version was reported to be acceptable [10]. The US-DES scale has been translated into other languages such as Chinese and Swedish, and the psychometric analysis has supported the validity and reliability of the new versions similarly [11,12].

In order to evaluate our empowerment education programs, we likewise need an Iranian version of DES. Therefore, the aim of this study is to assess the psychometric properties of the Iranian version of the Diabetes Empowerment Scale.

## Methods

### Procedure

This study was conducted in two stages:

1- The preparation of the Persian version of DES-28: after translating the English version of DES-28 by 3 bilingual translators and thereafter back translating it, the questionnaire was studied by clinicians and patients both of them involved in the field of diabetes. The questionnaire was then modified based on their opinions.

2- Psychometric evaluations: The last Persian version of DES-28 was distributed among the participants, who had given an informed consent, in two stages (at the beginning of the study and then two months later). The patients then completed the questionnaires while they were seated in a comfortable and quiet room. The DES questions were read to illiterate participants by the physician and their answers were recorded.

### Subjects

The present cross sectional study was conducted in diabetes clinic of Shariati hospital, Tehran University of Medical Sciences (TUMS). One hundred patients were selected from among 4500 patients who had a medical record in the clinic using a random sampling method. Patients with type 2 diabetes, who aged more than 18 years and had no serious complication, such as unstable coronary artery disease, severe heart failure, stroke with sequel, end-stage renal disease, severe peripheral vascular disease and severe psychiatric disorders including dementia and schizophrenia that affects the patients’ cognitive ability were enrolled.

The ethic committee of TUMS approved this study and all patients signed informed consent.

### Measurements

We used the 28-item US version of DES with three subscales, including managing the psychosocial aspects of diabetes, assessing dissatisfaction and readiness to change, and setting and achieving diabetes goals [10]. A five-point scale was used to answer the items. The responses ranged from strongly agree (5 points) to strongly disagree (1 point) and therefore higher scores indicated better adjustment to the illness. In addition to DES, the third versions of the Diabetes Attitude Scale (DAS-3) and the Problem Areas in Diabetes (PAID) were also filled out by the patients. The third version of DAS developed in 1998, consists of 33 Likert scale items (5=strongly agree, 1=strongly disagree) and measures five discrete subscales: need for special training, seriousness of type 2 diabetes, value of tight control, psychosocial impact of diabetes, and patient autonomy. They were also answered using a five-point scale, ranging from strongly agree to strongly disagree [13]. The PAID scale is a self-administrated questionnaire that consists of twenty eight statements identified as common emotional problems related to living with diabetes. The questionnaire assesses four domains of diabetes-related quality of life: emotional distress, treatment barriers, problems related to food and lack of social support [14,15]. Each item can be rated using a 5-point Likert scale, ranging from 0 to 4. The final score is calculated after multiplying the total score, resulted in through summing the 0–4 responses given for each of the 20 items, by 1.25. Higher scores indicate greater emotional distress [16]. The original version of the scale has proved its validity and reliability [15], also in other studies indicated that The PAID scale has good psychometric properties [17-19]. Moreover, a self report questionnaire was used to record the demographic characteristics (gender and age) along with data on his/her diabetes treatment, education, and duration of diabetes) in each patient. Current HbA_1c_ values were extracted from the patients’ medical records.

### Statistical analysis

Data were entered and analyzed using Statistical Package for the Social Sciences (SPSS-16). The reliability of the questionnaire was evaluated using internal consistency (total Cronbach’s alpha (α) and α for each subscales), test- retest and splitting methods (spearman-brown coefficient correlation). Also the validity of the questionnaire was evaluated by construct validity (scores test, varimax rotation, keiser-meyer-olkin measuring of the samples and Bartlett’s test of Sphericity), criterion validity (the correlation between the DES total score and extracted factors with the duration of diabetes was assessed by Spearman correlation due to the lack of normal distribution and the correlation between DES total score and extracted factors with HbA_1c_ was calculated by Pearson correlation) and concurrent validity (correlation between the scores obtained in DES and that of PAID and DAS).

## Results

### Demographic and clinical data

Totally 100 patients (male=54, female=46) with type 2 diabetes were interviewed. The mean age of the participants was 52.65 (SD=7.1) years. The average duration of diabetes in the studied patients was 8.64 (SD=5.80) years and their mean HbA_1c_ value was equal to 6.86 (SD=0.37). Twenty one of the participants were illiterate. Twenty one of the participants were illiterate. Fifty nine subjects used oral agents, 11 were on insulin and 30 received both oral agents and insulin (Table 1).

**Table 1:** Demographic and clinical data

***Characteristics***	**%**
**Sex**	
Men	54
women	46
Age^*^	52.65 (7.1)
Duration of diabetes (years)^*^	8.64 (5.80)
HbA_1c_^*^	6.86 (0.37)
**Education**	
Illiterate	21
Elementary school	14
High school	47
University	18
**Diabetes treatment**	
Insulin	11
Oral agents	59
Insulin and Oral agents	30

^*^ Data for the age, duration of diabetes and HbA1c are mean (SD). (n=100).

### Reliability

The α-coefficient for IR-DES-28’s total score was 0.89 and for its three subscales (calculated by factor analysis) was 0.80, 0.50, and 0.79, respectively. The results showed a high internal consistency for the questionnaire. The total test-retest reliability was 0.74 (P <0.01). Moreover splitting method showed the instrument to be completely reliable (correlation between the forms=0.73, first part α =0.80, the second part α =0.84 and reliability coefficient=0.82).

### Validity

#### Construct validity

The Keiser-Meyer-Olkin measure of sampling (Kmo=0.77) and Bartlett’s test of Sphericity (Bts=1197) indicated that the sample size was suitable for conducting a factor analysis and the correlation matrix has not occurred by chance. The result suggested seven factors for this scale. The method of factor analysis was least factor load. After an iterative factor processing and item analysis, a three-factor solution was judged the best (Table 2). The variance for each factor was reported to be 27.62, 21.65, and 12.65 percent, respectively. The total variance was equal to 61.52% and all the three factors were shown to have eigenvalues higher than 1.0. A list of IR-DES-28 items and its subscales are displayed in Table 3.

**Table 2:** Results of psychometrics indicators of factor analysis of IR-DES-28 (n=100)

**Factor**	**Eigenvalue**	**Communalities**	**Variance%**	**Cumulative%**	**N. of questions**
first	7.73	0.68	27.62	27.62	12
second	2.29	0.53	21.65	49.27	4
third	2.03	0.72	12.25	61.52	12

**Table 3:** Items of the three subscales of IR-DES-28

**Subscale name**	**Items**
	**In general, I believe that I:**
Self-awareness and	…know what part(s) of taking care of my diabetes that I am satisfied with.
managing Psychological	…know what part(s) of taking care of my diabetes that I am dissatisfied with.
aspects of diabetes	…can tell how I’m feeling about having diabetes.
	…know the positive ways I cope with diabetes-related stress.
	…can cope well with diabetes- related stress.
	…know where I can get support for having and caring for my diabetes.
	…can ask for support for having and caring for my diabetes when I need it.
	…can support myself in dealing with my diabetes.
	…know what helps me stay motivated to care for my diabetes.
	…know enough about diabetes to make self-care choices that are right for me.
	…know enough about my- self as a person to make diabetes care choices that are right for me.
	…am able to figure out if it is worth my while to change how I take care of my diabetes.
Goal achievement ability	…can choose realistic diabetes goals.
	…know which of my diabetes goals are most important to me.
	…can reach my diabetes goals once I make up my mind.
	…know which barriers make reaching my diabetes goals more difficult.
The ability of setting goals	…know what part(s) of taking care of my diabetes that I am ready to change.
	…know what part(s) of taking care of my diabetes that I am not ready to change.
	…know the things about myself that either help or prevent me from reaching my diabetes goals.
	…can come up with good ideas to help me reach my goals.
	…am able to turn my diabetes goals into a workable plan.
	…can think of different ways to overcome barriers to my diabetes goals
	…can try out different ways of overcoming barriers to my diabetes goals.
	…am able to decide which way of overcoming barriers to my diabetes goals works best for me.
	…can tell how I’m feeling about caring for my diabetes.
	…know the ways that having diabetes causes stress in my life.
	…know the negative ways I cope with diabetes-related stress.
	…can motivate myself to care for my diabetes.

#### Criterion validity

The existence of any association between HbA_1c_ levels, as the indicator of metabolic control and duration of diabetes, and the DES total score and its subscales was assessed and the result indicated the correlation between HbA_1c_ levels and IR-DES-28 total score and its subscales was significant (Table 4).

**Table 4:** Correlations between IR-DES-28 and diabetes-related data

**Factors**	**duration of diabetes**	**HbA**_**1c**_
Self-awareness and managing psychological aspect of diabetes	−0.05	−0.33^a^
Goal achievement ability	−0.1	−0.33^a^
Set goals ability	−0.08	−0.32^a^
Total	−0.09	−0.36^a^

Correlations were performed with Pearson’s correlation or Spearman method.

^a^ P <0.01.

#### Concurrent validity

The assessment of correlation between IR-DES-28 and IR-PAID-20 and IR-DAS-33 scales, indicated a negative correlation between overall DES-28 and PAID-20 scores (r=−0.21 and P <0.01) and a positive one between the overall DES-28 and DAS-33 scores (r=0.42 and P <0.01).

## Discussion

The present pilot study aimed to evaluate the validity and reliability of the Iranian Diabetes Empowerment Scale (IR-DES-28). It showed the validity and reliability of the DES, adding that IR-DES-28 has a high internal consistency, with overall Cronbach’s α coefficient of 0.89. Also the test-retest reliability and splitting method indicated the instrument to be completely reliable.

The factor analysis resulted in three factors with eigenvalues greater than 1.0. These three factors are as following:

Factor 1, “Self-awareness and managing psychological aspects of diabetes,” includes 12 items on self knowledge, positive and negative coping methods with diabetes-related stress.

Factor 2, “Goal achievement ability,” includes 12 items on having knowledge and the ability to achieve diabetes goals.

Factor 3, “the ability of setting goals,” is about having knowledge and the ability to determine diabetes goals.

In this study, the factor analysis showed a resemblance between the US and the Iranian version of the questionnaire, as both questionnaires yielded three subscales [10]. However the number of questions in each subscale differs between the two tools. In addition, the last C-DES-20 [11] and SW-DES-23 [12] versions each had five and four subscales, respectively, pointing out a discrepancy between the Iranian with the Swedish and Chinese versions.

While many of the previous studies have not reported any significant correlation between metabolic control and diabetes empowerment [12], the present study showed a mild negative correlation between these variables. This finding confirms the criterion validity of the IR-DES-28.

The assessment of concurrent validity between IR-DES-28 and IR-PAID-20 and IR-DAS-33 also confirmed based on our findings indicates a negative correlation between overall DES and PAID scores (r=−0.21 and P <0.01) consistent with the study conducted in Iceland [18] and a positive correlation between overall DES and DAS scores (r=0.42 and P <0.01). This simply shows that becoming empowered in diabetes management is associated with experiencing less diabetes-related psychological problems and adopting a better attitude towards diabetes, all of which would result in an enhanced diabetes management.

This study demonstrated psychometric properties such as reliability and validity of the Iranian version of DES scale was acceptable, these findings are consistent with previous studies [11,12].

## Competing interests

The authors declare that they have no competing interests.

## Authors’ contributions

BL, SMA, and RH conceived and designed the study and acquired funding. MYM and PKH collected data. RH conducted analysis. MYM and ENE drafted the manuscript. SMA and PKH edited the final article. The All authors provided critical revisions to the manuscript and made substantive intellectual contributions to the study. All authors read and approved the final manuscript.
